# Evaluation of the Endothelial Cell Density and the Central Corneal Thickness in Pseudoexfoliation Syndrome and Pseudoexfoliation Glaucoma

**DOI:** 10.1155/2014/123683

**Published:** 2014-07-01

**Authors:** Bożydar T. Tomaszewski, Renata Zalewska, Zofia Mariak

**Affiliations:** Department of Ophthalmology, Medical University of Bialystok Clinical Hospital, Medical University of Bialystok, M. Skłodowskiej-Curie 24 A, 15-276 Bialystok, Poland

## Abstract

*Purpose.* Evaluation of central corneal thickness (CCT) and endothelial cell density (ECD) in patients with senile cataract and coexisting pseudoexfoliation (PEX) syndrome with glaucoma (PEXG) and without glaucoma using specular microscopy.* Participants and Methods.* The study included 122 patients (217 eyes). In this group of patients we identified 133 eyes with PEX syndrome (65 with glaucoma, 68 without glaucoma) and 84 eyes without PEX syndrome. ECD and CCT were measured in each eye by specular microscopy.* Results.* ECD in eyes with PEX syndrome without glaucoma (2297 ± 359 cell/mm^2^) and in eyes with PEXG (2241 ± 363 cell/mm^2^) was lower than in the control group (2503 ± 262 cell/mm^2^) (*P* < 0.001). CCT in eyes with PEXG (508.2 ± 32.6 *μ*m) was thinner than in eyes with PEX syndrome without glaucoma (529.7 ± 30.3 *μ*m) and control group (527.7 ± 29.4 *μ*m) (*P* < 0.001).* Conclusions. *This research shows that in eyes with PEX syndrome, both with and without glaucoma, ECD was statistically significantly lower than in the control group. In patients with PEXG, CCT was statistically significantly thinner than in the PEX syndrome and control group.

## 1. Introduction

The pseudoexfoliation syndrome (PEX) is a systemic, age-related disorder with a strong genetic component [1–5]. It is characterized by the production and accumulation of extracellular granular amyloid-like material in many tissues and organs [6, 7]. A typical sign of the PEX syndrome in the eyeball, visible during an examination with the slit lamp biomicroscope, is white deposits accumulating on the pupillary border and on the anterior lens capsule [8]. Deposits of the pseudoexfoliative material can also be found on the inner layer of the ciliary epithelium, on the zonules of Zinn, on the iris epithelium, in the anterior chamber angle structures, and in the front part of the vitreous [3, 9]. Deposits of the PEX material can also take the form of irregular clumps on the corneal endothelium. These changes are closely related with cataract, glaucoma, and lens subluxation, pseudouveitis, retinal vein occlusion, and keratopathy [10].

The corneal endothelium is a single layer of hexagonal cells that do not have the ability to regenerate. The normal density of corneal endothelial cells in adults is approximately 2500 cells/mm^2^ and it is reduced by about 0.6% a year. The endothelium performs an essential function of maintaining the hydration of the cornea. When the endothelial cells density is reduced to approximately 800 cells/mm^2^, it may lead to corneal decompensation causing corneal edema and loss of corneal transparency, which disrupts vision [11].

The mean normal corneal thickness is approximately 542 *μ*m. Clinical studies have found that thicker corneas lead to overestimations and thinner corneas lead to underestimations in intraocular pressure (IOP) readings. It has been found that any 10 *μ*m deviation from the mean normal corneal thickness results in 0.5 mmHg difference in measurement when using a Goldmann tonometer [12]. Thus, it is possible to underestimate the IOP reading in the PEX syndrome and overlook an early glaucomatous damage. This could have serious clinical implications, since PEXG, which constitutes approximately 30% of PEX syndrome cases in Poland, shows faster progression of the optic disc damage and poorer prognosis in terms of preserving eyesight as compared to primary open-angle glaucoma.

The aim of this study was to assess central corneal thickness (CCT) and endothelial cell density (ECD) in patients with senile cataract and coexisting PEX syndrome with and without glaucoma using specular microscopy.

## 2. Patients and Methods

We examined 217 eyes of 122 patients with diagnosed senile cataract who were admitted to the Bialystok Ophthalmology Clinic during the years 2010–2013 for the surgical treatment of cataracts. Exclusion criteria included a history of previous eye surgery, glaucoma without PEX syndrome, refractive error greater than the absolute value of 2.0 D, a history of eyeball trauma, and any other corneal diseases.

The study was approved by the bioethical committee in Bialystok Medical University and was performed in accordance with ethical standards laid down in the 1964 Declaration of Helsinki. All patients signed a consent form before their inclusion in the study.

The patients were divided into 3 groups; group PEX consisted of 39 patients with senile cataract and PEX syndrome without glaucoma (we examined 68 eyes—37 corneas in 21 men and 31 corneas in 18 women), group PEXG included 37 patients diagnosed with PEXG (we examined 65 eyes—33 corneas in 19 men and 32 corneas in 18 women), and group CNT—the control group, consisted of 46 patients with senile cataract without coexisting PEX syndrome (we examined 84 eyes—46 corneas in 25 men and 38 corneas in 21 women). We excluded 10 eyes from group PEX due to previous cataract surgery (we examined only 1 eye in 10 patients). We excluded 9 eyes from group PEXG due to previous cataract surgery (we examined only 1 eye in 9 patients). We excluded 8 eyes from the control group due to previous cataract surgery (we examined only 1 eye in 8 patients).

While qualifying patients for individual study groups PEX syndrome was diagnosed on the basis of typical symptoms visible during an examination with a slit lamp biomicroscope. Every patient was examined by two independent doctors.

The main criterion for the qualification of patients into the PEXG group, excluding previous diagnosis and treatment of PEX glaucoma, was the cup disc ratio assessment. Any value exceeding 0.5 was considered to be an injury caused by glaucoma.

There were no patients whose eyes could qualify for two different study groups.

The mean age in group PEX was 76.9 ± 6.54 years (age range 61–93), group PEXG was 78.22 ± 7.58 years (age range 55–94), and the control group 76.65 ± 7.62 years (age range 61–91). No significant statistical difference (*P* > 0.1) between sex and age of patients of any study group has been shown (Table 1).

All patients underwent a complete eye examination, including evaluation of visual acuity for distance and near vision using Snellen chart, IOP measurement using Goldmann applanation tonometry, and slit-lamp examination of the anterior eye structures with assessment of the fundus of the eye performed with Volk aspheric Wide Field lens. Patients with significant ocular media opacity were examined with B-scan ultrasonography.

In all patients, ECD and CCT were assessed by specular microscopy with the use of Topcon SP-3000P. Specular microscopy is a noninvasive method to visualize and analyze the corneal endothelial cells. The image of endothelium is obtained when the instrument captures the light reflected from the optical interface between the corneal endothelium and the aqueous humor. Modern specular microscopes use advanced computer software to analyze the size, shape, and density of the endothelial cells and allow for the measuring of the central corneal thickness (pachymetry). In many comparison studies specular microscopy, especially model Topcon SP-3000P, has been defined to be more accurate and more reliable than the more common ultrasound pachymetry (USP) [13–15].

**Figure 1:**
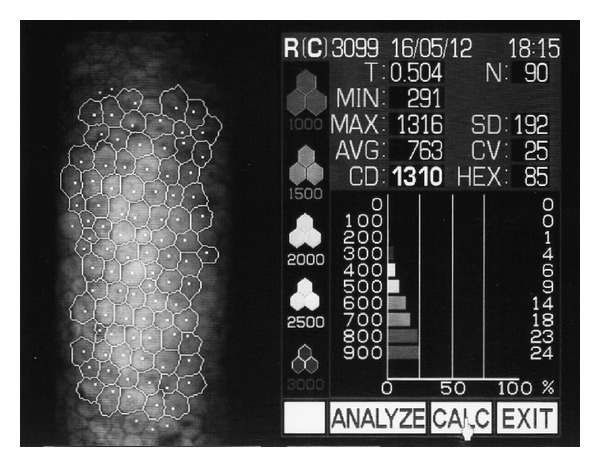
Specular photomicrograph from Topcon SP-3000P specular microscope. N: number of cells; T: central corneal thickness; MIN, MAX, AVG: minimum, maximum, and average size of cell area; CD: cell density; SD: standard deviation, CV: coefficient of value; HEX: hexagonal cell ratio.

The examination was carried out in automatic mode. In order to achieve the most accurate measurement of density, at least 60 adjacent cells were manually selected on the specular photomicrograph of a 0.5 × 0.25 mm section of endothelial surface (Figure 1). Three microphotographs were performed for every eye (the difference of ECD and CCT values did not exceed ±5%). Average ECD and CCT values were used in further calculations. Following that, the device performed an automatic analysis of the selected area and calculated the average number of cells per 1 mm^2^ and the CCT in mm. The microscope then provided a histogram determining the endothelial cell population size and specified the minimum, maximum, and average cell size of the selected area. The pleomorphism of endothelial cells was also evaluated, indicating the percentage of hexagonality. Statistical analysis of the calculations determined the standard deviation and the coefficient of variation.

Further statistical analysis was performed using computer software Statistica v. 8.0 (StatSoft, USA). Arithmetic mean and standard deviation of quantitative properties being considered were calculated for every group. The chi-squared test was performed for qualitative properties. We verified the character of ECD and CCT distribution in patients from group I, group II, and the control group. Using Shapiro-Wilk, Lilliefors, and Kolmogorov-Smirnov tests we found that the random cell sample originated from a cell population with normal distribution of variables of mean ECD and CCT. Levene and Brown-Forsythe tests showed no significant differences in variances of the studied groups. We used the ANOVA test to exclude the hypothesis that the means in the studied groups are equal. To compare the differences between the studied groups we used Tukey's post hoc test, considering *P* < 0.05 as statistically significant.

## 3. Results

**Figure 2:**
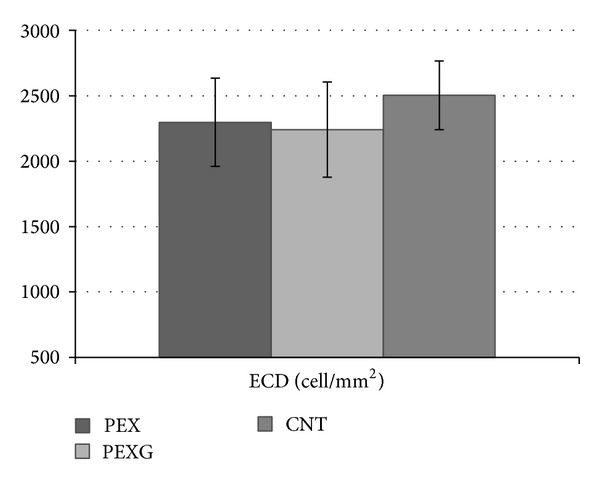
Evaluation of cell density (ECD) in groups of patients with PEX syndrome (PEX), PEX glaucoma (PEXG), and the control group (CNT).

In group PEX, ECD (2297 ± 359 cells/mm^2^) was lower than in the control group (2503 ± 262 cells/mm^2^) with statistical significance at *P* = 0.0008. In group PEXG, ECD (2241 ± 363 cells/mm^2^) was also lower than in the control group with statistical significance at *P* = 0.000005. The difference between ECD in group PEX and group PEXG (*P* = 0.77) was not statistically significant (Table 2, Figure 2).

**Figure 3:**
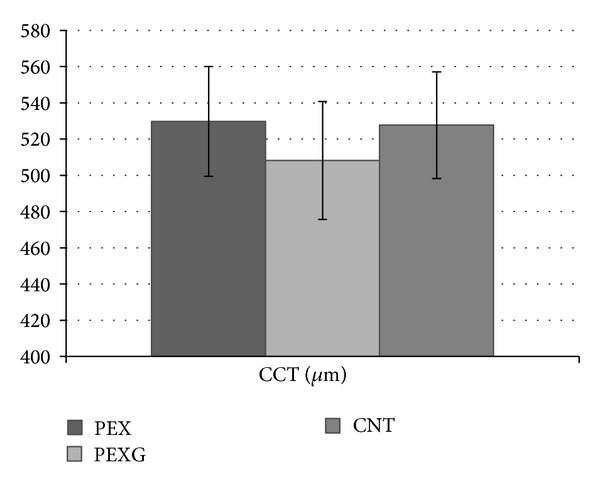
Evaluation of central corneal thickness (CCT) in groups of patients with PEX syndrome (PEX), PEX glaucoma (PEXG), and the control group (CNT).

In group PEX, CCT (529.7 ± 30.3 *μ*m) did not differ from the control group (527.7 ± 29.4 *μ*m) with statistical significance at *P* = 0.912. In group PEXG, CCT (508.2 ± 32.6) was statistically significantly thinner than in group PEX and control group (CNT) (resp., *P* = 0.00017; *P* = 0.00036) (Table 2, Figure 3).

## 4. Discussion

We used specular microscopy to compare ECD and CCT in patients with PEX syndrome, with PEXG, and in the control group. Patients were admitted to the Department of Ophthalmology, Medical University of Bialystok Clinical Hospital, for senile cataract surgery.

### 4.1. ECD

The analysis of the optical density of the endothelium of main study group was a significant part of the conducted study. Statistical calculations demonstrated that the lowest cell density of the endothelium occurred in eyes of patients from the PEXG group (2241 ± 363 cells/mm^2^). The value was slightly higher for patients with PEX syndrome without glaucoma (2297 ± 359 cells/mm^2^); however, no significant statistical variance has been shown for these groups (*P* = 0.77). In the control group the cell density of the endothelium was the highest (2503 ± 262 cells/mm^2^) and was significantly different from the values obtained for the other study groups (*P* < 0.001).

During our analysis of literary data from all over the world dealing with ophthalmology we have not encountered any attempt to simultaneously compare the PEX, PEXG, and control study groups.

Research presented by Inoue et al., Seitz et al., and Wang et al. shows that the cell density of the endothelium of the PEX group (without considering glaucoma) was lower than that of the control group. Through further analysis the authors found that patients with PEX syndrome and secondary glaucoma have a lower endothelial cell density than people with PEX syndrome without glaucoma (Table 3) [16–18].

The authors compared the cell density of the corneal endothelium of patients with PEX syndrome, disregarding the presence of secondary glaucoma, to the control group. During the next stage patients were separated into groups of those with PEX syndrome and secondary glaucoma and those without secondary glaucoma with the groups being subsequently compared to one another. These two subgroups, however, were not compared to the control group. In all probability this resulted from the very small number of patients' eyes with PEX syndrome but without glaucoma (resp., 16, 7, and 19 eyes). This factor could also be relevant for the significant variance discovered between the endothelial cell density of the group of patients with PEX syndrome and the group of patients with secondary glaucoma and PEX in the study performed by Seitz et al.

Wali et al. studied endothelial cell density of groups of patients with PEX syndrome with glaucoma and PEX without glaucoma. Their observations were consistent with the results of this study and the research published by Wang et al. and Seitz et al. All researchers noticed that patients with PEX glaucoma have lower endothelial cell density than those with PEX syndrome without glaucoma (2438 ± 503.4 cells/mm^2^ versus 2483 ± 511.2 cells/mm^2^) but these results never reached statistical significance (*P* = 0.629) [19].

Authors of other studies only compared patients with PEX syndrome, without considering glaucoma, to the control group. All research presented a lower endothelial cell density for patients with PEX (Table 4) [20–23].

In reviewed scientific literature only Żarnowski et al. performed a comparison of endothelial cell density of patients with secondary glaucoma and PEX to the control group. The results obtained in this study confirmed that patients with PEXG have a lower endothelial cell density than healthy people (2128 ± 483 cells/mm^2^ versus 2753 ± 354 cells/mm^2^; *P* < 0.001) [23].

Research presented above clearly shows that PEX syndrome significantly influences cell density of corneal endothelium of people with this disease. The cause of lower endothelial cell density of patients with PEX syndrome is the pseudoexfoliation material, appearing at the earliest stages of PEX, which settles on the endothelium penetrating it in the direction of the Descemet's membrane and breaking the connections between individual six-sided cells, which results in local accelerated apoptosis of these cells. Other factors recognized by researchers, excluding the accumulation of PEX material causing the reduction of the number of cells within the layer of the corneal endothelium, include hypoxia of the anterior chamber, changes in the fibroblasts of the endothelium, and elevated concentration of TGF-*α*1 [5, 24]. The simultaneous occurrence of glaucoma further intensifies and accelerates the deterioration of endothelial cells. Reduction of endothelial cell density to less than 800 cells/mm^2^ results in corneal decompensation—the endothelium ceases to seal the cornea allowing liquids to seep into the corneal stroma causing irreversible swelling and loss of translucency [25]. In dramatic circumstances the patient suddenly loses his sight.

In order to predetermine which patients possess an elevated risk of corneal decompensation a simple index based on endothelial cell density has been implemented during routine intraocular procedures. The value of 2000 cells/mm^2^ has been established as a reference line and patients whose endothelial cell density is lower than 2000 cells/mm^2^ are considered to be high risk patients.

Over 20% of examined patients with PEX syndrome, regardless of presence of glaucoma, had an elevated risk of corneal endothelium decompensation (Table 5).

Quiroga et al. also showed that in their group of patients with PEX syndrome 21.3% corneas had endothelial cell density below 2000 cells/mm^2^ [21].

The facts presented above should be considered during preparation of patients, especially older patients with PEX glaucoma, for antiglaucoma surgical procedures and typical cataract removal operations using the phacoemulsification method. It has been proven by the authors that after intraocular surgery loss of endothelial cell density oscillates between 6% and 19% one year from the date of the procedure [11, 26–28]. To reduce this phenomenon the endothelium should be protected through the use of dispersive or adaptive viscoelastic substances to maintain proper depth of the anterior chamber and to prevent surgical instruments from coming in contact with the endothelium and to limit the depressive effect of ultrasonic waves on this structure.

### 4.2. CCT

The results obtained prove that the thinnest corneas occur in eyes of patients with secondary glaucoma with PEX (508.2 ± 32.6 *μ*m) and this value is different in a statistically significant way (*P* < 0.001) from the central thickness of the cornea of patients with PEX syndrome but without glaucoma (529.7 ± 30.3 *μ*m) and from the CCT of people from the control group (527.7 ± 29.4 *μ*m). Additionally, it has been shown that patients with PEX syndrome but without glaucoma had the thickest corneas; however, in comparison with the control group this variance was not statistically significant (*P* = 0.912).

Those few study results published in scientific literature assessing the effect of the PEX syndrome on central corneal thickness have been ambiguous.

Results which have been consistent with the conclusions of this study have been presented by Kitsos et al, where an ultrasonic pachymeter was used for the assessment of central corneal thickness [29]. The authors showed that the lowest central corneal thickness measurement value was obtained in patients with PEX glaucoma (526.00 ± 34.30 *μ*m) and this value differed in a statistically significant way from the central corneal thickness of patients with PEX syndrome (550.64 ± 39.0 *μ*m) and patients of the control group (547.36 ± 33.1 *μ*m) (*P* < 0.05). Despite the fact that the study presented the central corneal thickness of patients with PEX syndrome without glaucoma as being the highest, in comparison to the control group these differences were not statistically significant.

Different data was presented by Inoue et al. [16]. According to the authors of this study patients with PEX syndrome with glaucoma and without secondary glaucoma had thinner corneas (529 ± 31 *μ*m) from the control group (547 ± 28 *μ*m) (*P* = 0.03). At the same time the researchers noticed that patients with secondary glaucoma and PEX had thicker corneas (534 ± 37 *μ*m) than people with PEX syndrome but without secondary glaucoma (528 ± 29 *μ*m); however, statistical significance was not achieved in the comparison between these two groups (*P* = 0.68). The discrepancy in the results presented in relation to the conclusions of this study may be a consequence of a very small group of participants used by Inoue et al.—19 corneas of patients with PEX syndrome and only 7 with secondary glaucoma and PEX were included in this study.

Authors of remaining studies measuring CCT compared results between two study groups: the control group and a group of patients with PEX syndrome without consideration for secondary glaucoma or the control group and patients with glaucoma and PEX.

By contrasting research comparing central corneal thickness of people with PEX syndrome without considering secondary glaucoma with patients without PEX we can see that the thickness of the cornea of patients with PEX syndrome is greater than of the people from the control group. Data consistent with this was presented by Hepsen et al. (PEX = 546.6 ± 39.6 *μ*m versus NoPEX = 542.9 ± 32.2 *μ*m) and Arnarsson et al. (PEX = 533 ± 32 *μ*m versus NoPEX = 527 ± 40 *μ*m). The variance was not statistically significant (*P* = 0.56; *P* = 0.232, resp.) [30, 31].

Results obtained by Acar et al. presented a lower corneal thickness in patients with PEX syndrome (540.8 ± 30.2 *μ*m) in comparison with the control group (551.5 ± 28.3 *μ*m). No statistical significance was achieved (*P* = 0.315) [32].

On the basis of the studies conducted by Hepsen et al, Arnarsson et al., and Acar et al. it is difficult to conclude with any precision whether central corneal thickness of people with PEX syndrome is higher or lower than the CCT of people without PEX syndrome. None of the studies mentioned previously showed statistically significant variance and the final values of CCT of patients with PEX could have been influenced by the large percentage of people with secondary glaucoma and PEX.

The literature reviewed concerned with the comparison of central corneal thickness of patients with secondary glaucoma and PEX in relation to the group of people without glaucoma and without PEX confirms the conclusions reached by this study.

Shah et al. proved that patients with glaucoma PEX had thinner corneas than people with PEX without glaucoma (530.7 *μ*m versus 553.9 *μ*m) recording a variance in statistical significance reaching a level of *P* < 0.001 [33]. Yagci et al. and Sobottka et al. also noticed that people with PEXG had thinner corneas than the patients of the control group (526.28 ± 31.73 *μ*m versus 533.96 ± 29.25 *μ*m; 507 ± 25 *μ*m versus 524 ± 25 *μ*m resp.). The authors however did not show a variance which was statistically significant (*P* > 0.05) [34, 35]. This may result from the fact that the groups of patients with secondary glaucoma included in their studies were quite small (Yagci et al., 25 PEXG corneas, Sobottka et al., 13 PEXG corneas). Interesting conclusions regarding the influence of PEX syndrome on the corneal stroma were included in the study published by Zheng et al. The authors, using confocal microscopy, identified deposits of pseudoexfoliating materials in the cornea itself. They also showed that the number of keratocytes in the corneal stroma of the eyes of patients with PEX syndrome (per unit of area) was smaller than in the group of people without PEX. They concluded that the presence of the pseudoexfoliating material induces apoptosis of corneal stroma keratocytes and in the end leads to the impoverishment of its extracellular structure [20]. This may result in the thinning of the cornea and its greater susceptibility to elevated intraocular pressure.

This fact is confirmed by the research of this study which concludes that corneas of patients with PEX glaucoma are the thinnest. Therefore, it should be accepted that the hydrodynamic forces exerting constant pressure on the walls of the eyeball, with its greater susceptibility to intraocular pressure, cause a reduction of corneal thickness.

## 5. Conclusions

This research shows that in eyes with PEX syndrome, both with and without glaucoma, ECD was statistically significantly lower than in the control group, which may increase the risk of corneal decompensation after intraocular surgeries. No statistically significant difference was found between ECD in the group of patients with PEX syndrome and the PEXG group.

In patients with PEXG, CCT is thinner than in the PEX syndrome and control group. However, no statistically significant difference was found in CCT of the PEX syndrome group versus the control group.

## Figures and Tables

**Table 1:** Patient demographics.

Groups	Number of patients		Age, y (Mean ± SD)	
PEX	39	F—18	76.49 ± 6.54	F—79.44 ± 5.24
		M—21		M—73.95 ± 6.59

PEXG	37	F—18	78.22 ± 7.58	F—77.06 ± 6.58
		M—19		M—79.32 ± 8.45

CNT	46	F—21	76.65 ± 7.26	F—79.67 ± 7.14
		M—25		M—74.12 ± 7.20

CNT: control group, PEX: pseudoexfoliation syndrome group, PEXG: pseudoexfoliation glaucoma group, F: female, M: male.

**Table 2:** Summary of results.

Groups	ECD	*P*	CCT	*P*
	(cells/mm^2^)		(*μ*m)	
CNT versus PEX	2503 ± 262 versus 2297 ± 359	**0.0008**	527.7 ± 29.4 versus 529.7 ± 30.3	0.912
CNT versus PEXG	2503 ± 262 versus 2241 ± 363	**0.000005**	527.7 ± 29.4 versus 508.2 ± 32.6	**0.00017**
PEX versus PEXG	2297 ± 359 versus 2241 ± 363	0.77	529.7 ± 30.3 versus 508.2 ± 32.6	**0.00036**

CNT: control group, PEX: pseudoexfoliation syndrome group, PEXG: pseudoexfoliation glaucoma group, ECD: cell density, CCT: central corneal thickness.

**Table 3:** Result summary of research comparing cell density of the corneal endothelium (ECD) of patients with PEX syndrome to the ECD of people without PEX syndrome and PEX to PEXG.

Authors	ECD PEX versus ECD CNT	*P*	ECD PEX versus ECD PEXG	*P*
	cells/mm^2^		cells/mm^2^	
Inoue et al. [16]	2336 ± 383 versus 2632 ± 327	**0.003**	2337 ± 407 versus 2332 ± 336	0.98
Seitz et al. [17]	2052 ± 264 versus 2372 ± 276	**<0.001**	2214 ± 251 versus 2014 ± 254	**0.008**
Wang et al. [18]	2298 ± 239 versus 2652 ± 18	**0.026**	2505 ± 284 versus 2186 ± 2	0.278

**Table 4:** Result summary of research comparing cell density of the corneal endothelium (ECD) of patients with PEX syndrome to the ECD of people without PEX syndrome.

Authors	ECD PEX	ECD CNT	*P*
	cells/mm^2^	cells/mm^2^	
Zheng et al. [20]	2240.7 ± 236.6	2738.7 ± 233.2	**<0.0001**
Quiroga et al. [21]	2315; SE = 49.13	2482; SE = 20.36	**0.002**
Kovaliunas et al. [22]	2228.57 ± 290.01	2500.96 ± 351.77	**0.003**
Żarnowski et al. [23]	2255 ± 299	2721 ± 352	**<0.001**

**Table 5:** The risk of corneal endothelium decompensation of individual study groups.

Groups	ECD ≤ 2000 cells/mm^2^	ECD > 2000 cells/mm^2^
	
PEX	**20.59%**	79.41%
PEXG	**23.08%**	76.92%
CNT	3.57%	**92.43%**

## Units, Symbols, and Abbreviations

ECD: Endothelium cell density
CCT: Central corneal thickness
PEX: Pseudoexfoliation
PEXG: PEX glaucoma
IOP: Intraocular pressure
D: Diopter
SE: Standard error
mm: Millimeter
*μ*m: Micrometer
mm^2^: Square millimeter.

## References

[1] Allingham RR, Loftsdottir M, Gortfredsdottir MS (2001). Pseudoexfoliation syndrome in Icelandic families. *British Journal of Ophthalmology*.

[2] Forsman E, Cantor RM, Lu A (2007). Exfoliation syndrome: prevalence and inheritance in a subisolate of the Finnish population. *Acta Ophthalmologica Scandinavica*.

[3] Kurowska A, Kaminska A, Izdebska J, Szaflik JP, Szaflik J (2009). Zespół pseudoeksfoliacji (PEX)-schorzenie ogólnoustrojowe. *Klinika Oczna*.

[4] Ritch R, Schlötzer-Schrehardt U (2001). Exfoliation syndrome. *Survey of Ophthalmology*.

[5] Schlötzer-Schrehardt U, Naumann GO (2006). Ocular and systemic pseudoexfoliation syndrome. *The American Journal of Ophthalmology*.

[6] Schumacher S, Schlötzer-Schrehardt U, Martus P, Lang W, Naumann GO (2001). Pseudoexfoliation syndrome and aneurysms of the abdominal aorta. *The Lancet*.

[7] Schlotzer-Schrehardt UM, Koca MR, Naumann GOH, Volkholz H (1992). Pseudoexfoliation syndrome: ocular manifestation of a systemic disorder?. *Archives of Ophthalmology*.

[8] Naumann GOH, Schlötzer-Schrehardt U (2000). Keratopathy in pseudoexfoliation syndrome as a cause of corneal endothelial decompensation. *Ophthalmology*.

[9] Ludwisiak-Kocerba L, Hevelke A, Kęcik D (2006). Zespół pseudoeksfoliacji—etiopatogeneza i objawy kliniczne. *Klinika Oczna*.

[10] Martone G, Casprini F, Traversi C, Lepri F, Pichierri P, Caporossi A (2007). Pseudoexfoliation syndrome: in vivo confocal microscopy analysis. *Clinical and Experimental Ophthalmology*.

[11] Bourne WM, McLaren JW (2004). Clinical responses of the corneal endothelium. *Experimental Eye Research*.

[12] Doughty MJ, Zaman ML (2000). Human corneal thickness and its impact on intraocular pressure measures: a review and meta-analysis approach. *Survey of Ophthalmology*.

[13] Tai L, Khaw K, Ng C, Subrayan V (2013). Central corneal thickness measurements with different imaging devices and ultrasound pachymetry. *Cornea*.

[14] Almubrad TM, Osuagwu UL, AlAbbadi I, Ogbuehi KC (2011). Comparison of the precision of the Topcon SP-3000P specular microscope and an ultrasound pachymeter. *Clinical Ophthalmology*.

[15] Ogbuehi KC, Osuagwu UL (2012). Repeatability and interobserver reproducibility of Artemis-2 high-frequency ultrasound in determination of human corneal thickness. *Clinical Ophthalmology*.

[16] Inoue K, Okugawa K, Oshika T, Amano S (2003). Morphological study of corneal endothelium and corneal thickness in pseudoexfoliation syndrome. *Japanese Journal of Ophthalmology*.

[17] Seitz B, Muller EE, Langenbucher A, Kus MM, Naumann GOH (1995). Endothelial keratopathy in pseudoexfoliation syndrome: quantitative and qualitative morphometry using automated video image analysis. *Klinische Monatsblätter für Augenheilkunde*.

[18] Wang M, Sun W, Ying L (2012). Corneal endothelial cell density and morphology in Chinese patients with pseudoexfoliation syndrome. *International Journal of Ophthalmology*.

[19] Wali UK, Al-Mujaini AS, Al-Kharusi NS, Bialasiewicz AA, Rizvi SG (2008). Quantitative and qualitative corneal endothelial morphology of omani patients with pseudoexfoliation syndrome. *Sultan Qaboos University Medical Journal*.

[20] Zheng X, Shiraishi A, Okuma S (2011). In vivo confocal microscopic evidence of keratopathy in patients with pseudoexfoliation syndrome. *Investigative Ophthalmology and Visual Science*.

[21] Quiroga L, van Lansingh C, Samudio M, Peña FY, Carter MJ (2010). Characteristics of the corneal endothelium and pseudoexfoliation syndrome in patients with senile cataract. *Clinical and Experimental Ophthalmology*.

[22] Kovaliunas E, Stech S, Jurkute N (2012). Characteristics of the corneal endothelium and pseudoexfoliation syndrome in patients with senile cataract. *Acta Ophthalmologica*.

[23] Żarnowski T, Łękawa A, Dyduch A (2005). Gęstość śródbłonka rogówki u chorych z jaskrą. *Klinika Oczna*.

[24] Naumann GO, Schlötzer-Schrehardt U, Küchle M (1998). Pseudoexfoliation syndrome for the comprehensive ophtalmologist. Intraocular and systemic manifestations. *Ophthalmology*.

[25] Olsen T, Eriksen JS (1980). Corneal thickness and endothelial damage after intraocular lens implantation. *Acta Ophthalmologica*.

[26] Dick HB, Kohnen T, Jacobi FK, Jacobi KW (1996). Long-term endothelial cell loss following phacoemulsification through a temporal clear corneal incision. *Journal of Cataract and Refractive Surgery*.

[27] Walkow T, Anders N, Klebe S (2000). Endothelial cell loss after phacoemulsification: relation to preoperative and intraoperative parameters. *Journal of Cataract & Refractive Surgery*.

[28] Bourne WM, Nelson LIL, Hodge DO (1997). Central corneal endothelial cell changes over a ten-year period. *Investigative Ophthalmology and Visual Science*.

[29] Kitsos G, Gartzios C, Asproudis I, Bagli E (2009). Central corneal thickness in subjects with glaucoma and in normal individuals (with or without pseudoexfoliation syndrome). *Clinical Ophthalmology*.

[30] Hepsen IF, Yağci R, Keskin U (2007). Corneal curvature and central corneal thickness in eyes with pseudoexfoliation syndrome. *Canadian Journal of Ophthalmology*.

[31] Arnarsson A, Damji KF, Sverrisson T, Sasaki H, Jonasson F (2007). Pseudoexfoliation in the Reykjavik eye study: prevalence and related ophthalmological variables. *Acta Ophthalmologica Scandinavica*.

[32] Acar BT, Buttanri IB, Sevim MS (2010). Evaluation of anterior segment parameters in pseudoexfoliation syndrome patients. *Turkish Journal of Ophthalmology*.

[33] Shah S, Chatterjee A, Mathai M (1999). Relationship between corneal thickness and measured intraocular pressure in a general ophthalmology clinic. *Ophthalmology*.

[34] Yagci R, Eksioglu U, Midillioglu I, Yalvac I, Altiparmak E, Duman S (2005). Central corneal thickness in primary open angle glaucoma, pseudoexfoliative glaucoma, ocular hypertension, and normal population. *European Journal of Ophthalmology*.

[35] Sobottka Ventura AC, Böhnke M, Mojon DS (2001). Central corneal thickness measurements in patients with normal tension glaucoma, primary open angle glaucoma, pseudoexfoliation glaucoma, or ocular hypertension. *British Journal of Ophthalmology*.

